# Genetic Epidemiology of Glucose-6-Phosphate Dehydrogenase Deficiency in the Arab World

**DOI:** 10.1038/srep37284

**Published:** 2016-11-17

**Authors:** C. George Priya Doss, Dima R. Alasmar, Reem I. Bux, P. Sneha, Fadheela Dad Bakhsh, Iman Al-Azwani, Rajaa El Bekay, Hatem Zayed

**Affiliations:** 1 Department of Integrative Biology, School of Biosciences and Technology, VIT University, Vellore, India; 2 College of Health Sciences, Biomedical Sciences Department, Qatar University, Doha, Qatar; 3 CIBER Pathophysiology of obesity and nutrition CB06/03, Carlos III Health Institute. Unidad de Gestion Clínica Intercentros de Endocrinología y Nutrición, Instituto de Investigación Biomédica de Málaga (IBIMA), Hospital Regional Universitario de Málaga/Universidad de Málaga, 29009, Málaga, Spain

## Abstract

A systematic search was implemented using four literature databases (PubMed, Embase, Science Direct and Web of Science) to capture all the causative mutations of Glucose-6-phosphate dehydrogenase (G6PD) deficiency (G6PDD) in the 22 Arab countries. Our search yielded 43 studies that captured 33 mutations (23 missense, one silent, two deletions, and seven intronic mutations), in 3,430 Arab patients with G6PDD. The 23 missense mutations were then subjected to phenotypic classification using *in silico* prediction tools, which were compared to the WHO pathogenicity scale as a reference. These *in silico tools* were tested for their predicting efficiency using rigorous statistical analyses. Of the 23 missense mutations, p.S188F, p.I48T, p.N126D, and p.V68M, were identified as the most common mutations among Arab populations, but were not unique to the Arab world, interestingly, our search strategy found four other mutations (p.N135T, p.S179N, p.R246L, and p.Q307P) that are unique to Arabs. These mutations were exposed to structural analysis and molecular dynamics simulation analysis (MDSA), which predicting these mutant forms as potentially affect the enzyme function. The combination of the MDSA, structural analysis, and *in silico* predictions and statistical tools we used will provide a platform for future prediction accuracy for the pathogenicity of genetic mutations.

G6PD is a housekeeping enzyme that is important in the pentose phosphate pathway (PPP) and essential for basic cellular function. G6PD also aids in producing compounds to prevent build-up of reactive oxygen species (ROS) within red blood cells^1^. The protein exists in both monomer and tetramer forms with the monomer consisting of 515 amino acids with a molecular weight of 59.625 kDa. The prevalence of G6PDD has been increasing through independent mutational events in different geographical areas where prevalence rate of malaria is currently or was previously endemic^2^. The clinical manifestations of G6PDD vary from asymptomatic individuals to patients with acute haemolytic anaemia, chronic non-spherocytic haemolytic anaemia, drug-induced haemolytic anaemia, favism, and neonatal jaundice^3^. G6PD mutations are classified by the WHO into five classes (Class I-V) according to their effect on the activity of the enzyme, where Class I is the severest, and Class V is normal^4^.

G6PDD is one of the most prevalent genetic diseases in the Arab countries; it is reported to have a high prevalence in Saudi Arabia (39.8%), Syria (30%), and Oman (29%) compared to other Arab countries^5^,^6^,^7^. More than 300 different mutations have been reported in the *G6PD* gene^8^; to date, the Human Gene Mutation Database (www.hgmd.cf.ac.uk/) has reported 202 mutations, with 68 pathogenic mutations that belong to Class I (WHO). The Mediterranean mutation (p.S188F) is the most prevalent among Arabs, with 90% frequency in Bahraini patients^9^,^10^,^11^, 87.8% in Northern Iraqi males, 74.2% in Kuwait, and 53.6% in Jordan^12^,^13^. Limited studies have investigated the incidence of G6PD mutations and their functional role in causing disease among Arab countries. In this study, we performed a systematic search to identify the mutations in the *G6PD* gene that are prevalent among Arab patients with GPDD. We found four mutations circulating among Arab populations that are shared with other ethnic groups, and four unique mutations that are distinctive to Arab populations. *In silico* prediction scores, structural analysis, and MDSA were performed to investigate the genotype-phenotype correlations in the patients harbouring these mutations. The results obtained from combination of different computational methods matched the WHO classification of these mutations, providing a practical evidence of the importance of the computational tools in predicting the effects of mutations on protein function.

## Results

**Figure 1:**
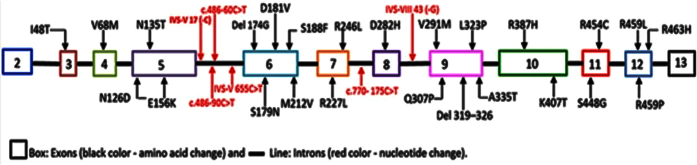
Mutations observed in the intronic and exonic regions of G6PD among the Arab populations.

**Figure 2:**
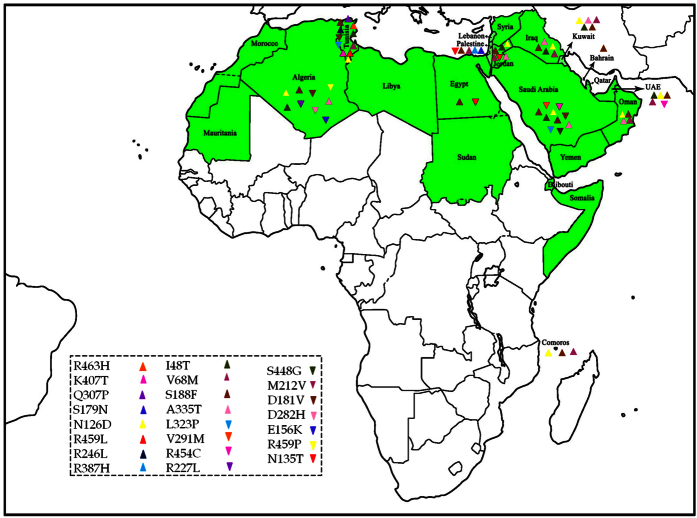
Distribution of missense mutations in the Arab world (green color) were marked in normal and inverted triangular symbols. The Map is created using the African continent (Africa’s regional Thumbnail) free map product (http://english.freemap.jp/item/africa/africa_1.html) licensed under the Creative Commons Attribution 3.0 unported (CC BY 3.0) license.

Our search strategy yielded 553 citations; of which 43 eligible articles were thoroughly screened and included in this study (Supplementary Fig. S1). A total of 3,430 Arab patients with G6PDD were captured, harbouring 33 mutations (23 missense mutations, one silent mutation, two deletions, and seven intronic mutations) (Figs 1 and 2). Tunisia, Saudi Arabia, and Jordan had most of the mutations circulated in the Arab countries. The 23 missense mutations were subjected to *in silico* prediction analysis to analyse the genotype-phenotype correlation of Arab patients with G6PDD (Supplementary Table S1). Out of the 23 missense mutations tested, 20, 18, 18, 17, and 18 were designated as disease (SNPs&GO), probably damaging (PolyPhen-2), deleterious (SIFT), effect (SNAP2), and functional impact (Mutationassessor), respectively. Various statistical parameters as shown in the methods were used to evaluate the performance of the 5 *in silico* prediction methods. Mutationassessor, SNPs&GO, PolyPhen-2 and SNAP2 scored best in terms of sensitivity/TPR with a score of 1. Of the five *in silico* methods, SNPs&GO was predicted with least FPR score of (0.33) which illustrates the better effectiveness in predicting the mutational effect as neutral. SNPs&GO (0.86) performed best in terms of accuracy followed by PolyPhen-2 & SIFT with a score of (0.78) and Mutationassessor & SNAP2 with a score of (0.74). All the predictions tools exhibited MCC value greater than 0, and none of the tools exhibited a negative value, indicating that the obtained results were more reliable and accurate. Overall observed results from the statistical analysis we conclude SNPs&GO as the best *in silico* tool in the prediction of deleterious mutations (Supplementary Table S2).

### Genotype-phenotype correlations

The frequency of the common mutations circulating among Arab patients with G6PDD was calculated by dividing the number of patients harbouring each mutation by the total number of patients, the p.S188F mutation was found to have highest prevalence among Arabs followed by p.N126D, p.V68M, and p.I48T mutations (Supplementary Table S3). These four mutations are not unique to Arabs; however, p.N135T, p.S179N, p.R246L, and p.Q307P mutations were identified as unique mutations to the Arab populations (Supplementary Table S1). Phenotypic classification using *in silico* tools were compared with the WHO pathogenicity reference scale to validate their prediction accuracy. Of the 23 missense mutations, 3 mutations were excluded as they were not reported by the WHO classifications. The rest 20 mutations are classified as 5% class I (severe), 55% class II (severe) and 40% class III (mild) by the WHO classification (Supplementary Table S1). The mutation classified as WHO class I (severe) was identified as pathogenic by SNPs&GO, PolyPhen-2, Mutationassessor, SNAP2 and SIFT. Among the 11 mutations classified as WHO Class II (severe); 10, 9, 8, 9 and 10 mutations were identified as pathogenic and the remaining as neutral by SNPs&GO, PolyPhen-2, Mutationassessor, SNAP2, and SIFT. Similarly for the 8 mutations classified as WHO Class III (mild); 6, 5, 5, 4 and 5 mutations were identified as pathogenic and the remaining as neutral by SNPs&GO, PolyPhen-2, Mutationassessor, SNAP2, and SIFT. These observed results conclude the tool SNPs&GO had higher percentage of matching towards both WHO Class II and Class III in predicting the mutations as disease causing or pathogenic. The common and unique Arab mutations were categorized and compared with the WHO classification as either Class II or Class III (Table 1). In conclusion, it seems to be a correlation between the *in silico* tool prediction scores and WHO reference scale in classifying the three mutations (common- p.S188F & unique- p.R246L, and p.Q307P) as Class II (severe), three common mutations p.N126D, p.V68M, and p.I48T as Class III (mild) and the remaining two unique mutations p.N135T and p.S179N as ‘not reported’.

### Conservation analysis

Sequence conservation analysis was performed primarily by building multiple sequence alignment (MSA) using Clustal Omega. The protein sequence of G6PD in humans is mostly conserved among different species, including mice, rat, hamster, bosin, and human (Supplementary Fig. S2). Subsequently, the obtained MSA was submitted as the input file to ConSurf tool to calculate the evolutionary conserved regions in common and unique mutational positions in G6PD (Supplementary Fig. S3). The observed results indicate amino acid position I48 as the highly conserved followed by V68, N126, and S188. When assessing the unique mutations, the amino acid positions N135 and S179 were not as highly conserved as the amino acids R246 and Q307. As a next step, we assessed the solvent accessibility property of each amino acid position using ConSurf results. In case of positions where common mutations are occurring, ConSurf predicted I48 and V68 in buried region and N126 and S188 in exposed region. Meanwhile, all four positions of unique mutations were observed in the exposed region may have functional effects as predicted by ConSurf (Supplementary Fig. S3).

### MDSA

The native and the 8 mutant protein structures were subjected to MDSA showed stabilization at 30 ns. To have a better uniqueness in the structural and functional analysis, simulation analysis of the trajectory files were compared between the common and unique mutations. The resultant trajectory files were used to analyse the changes in the protein structure and function.

### Common mutations

**Figure 3:**
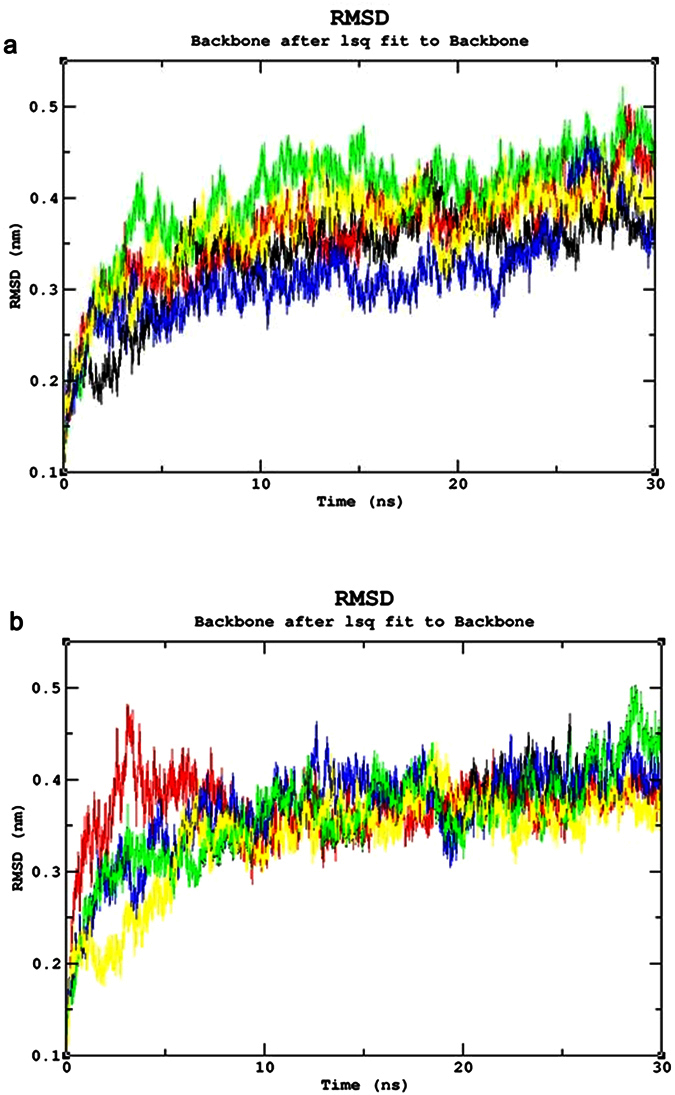
Root Mean Square Deviation (RMSD) Analysis (**a**) RMSD of the Common mutants and native. Color Scheme Native (Black), p.S188F (Red), p.N126D (Blue), p.V68M (Green), and p.I48T (Yellow). (**b**) Root Mean Square deviation of the unique mutants and native. Color Scheme Native (Black), p.S179N (Red), p.Q307P (Blue), p.R246L (Green), and p.S135T (Yellow).

**Figure 4:**
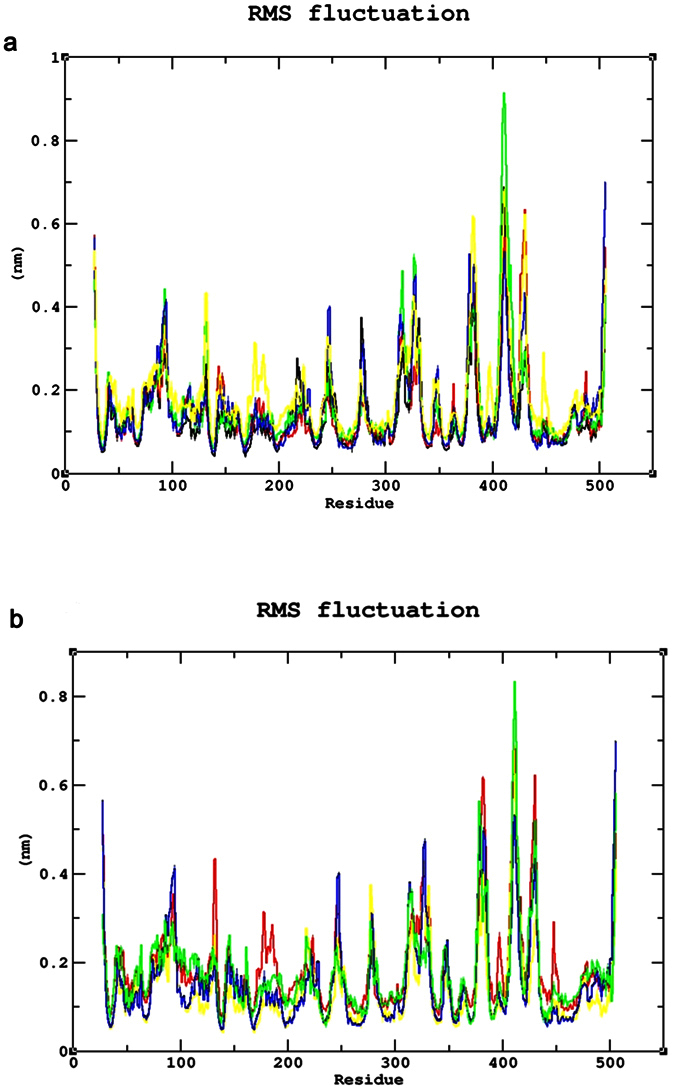
Root Mean Square Fluctuation (RMSF) analysis (**a**) RMSF of the common mutants and native. Color Scheme Native (Black), p.S188F (Red), p.N126D (Blue), p.V68M (Green), and p.I48T (Yellow). (**b**) RMSF of the unique mutants and native. Color Scheme Native (Black), p.S179N (Red), p.Q307P (Blue), p.R246L (Green), andp.S135T (Yellow).

**Figure 5:**
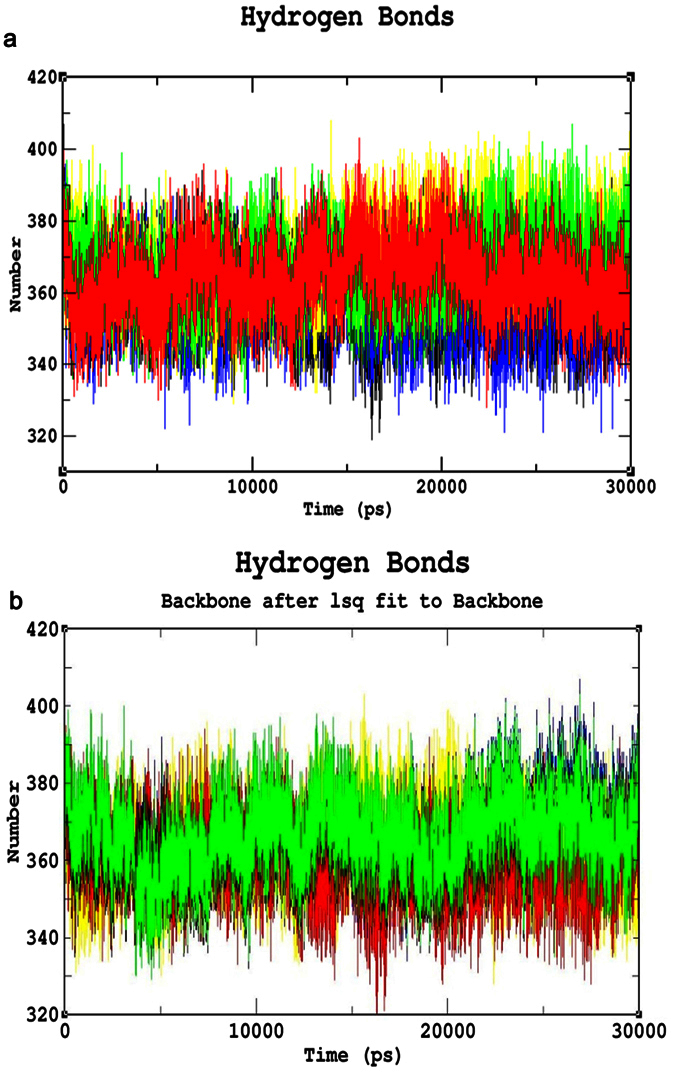
Hydrogen Bond Analysis (**a**) Hydrogen bond analysis of common mutants and native. Colour Scheme Native (Black), p.S188F (Red), p.N126D (Blue), p.V68M (Green), and p.I48T (Yellow). (**b**) Hydrogen bond analysis of the unique mutants and native. Color Scheme Native (Black), p.S179N (Red), p.Q307P (Blue), p.R246L (Green), and p.S135T (Yellow).

**Figure 6:**
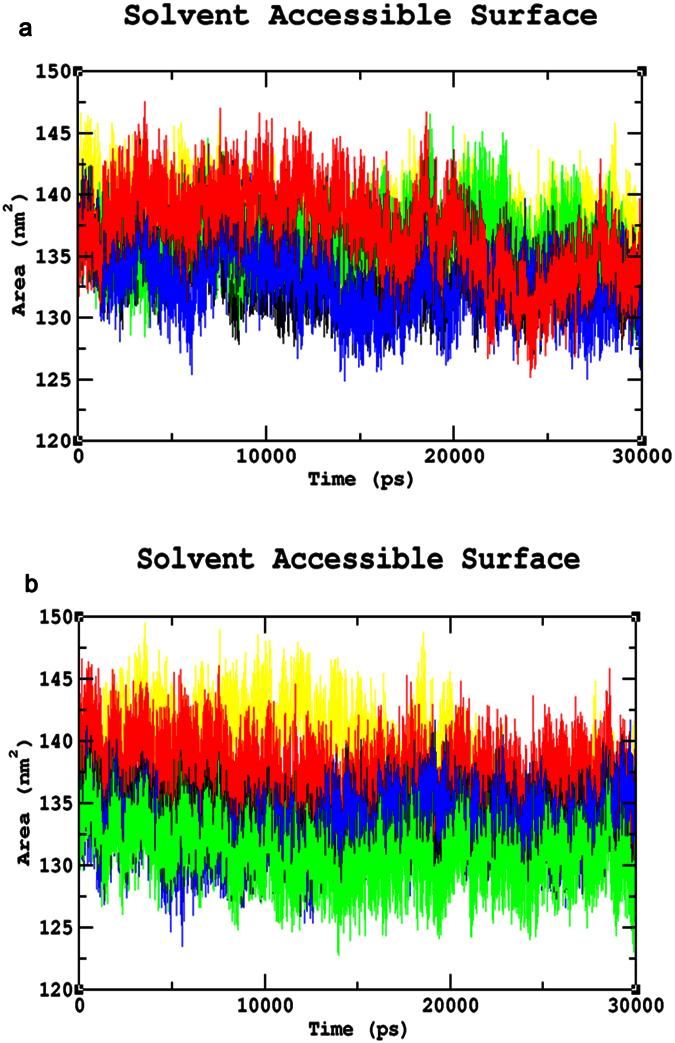
Solvent-Accessible Surface Area analyses (SASA) (**a**) SASA of Common mutants and native mutants. Color Scheme Native (Black), p.S188F (Red), p.N126D (Blue), p.V68M (Green), and p.I48T (Yellow). (**b**) SASA of the unique mutants and native. Color Scheme Native (Black), p.S179N (Red), p.Q307P (Blue), p.R246L (Green), and p.S135T (Yellow).

The Root Mean Square deviation (RMSD) results showed a large deviation pattern at the beginning of the simulation, which might be due to the initial strong kinetic shock experienced by the system. The native protein and mutant p.N126D showed comparable pattern of deviation between ~0.33 nm and ~0.35 nm, followed by the mutant p.I48T with slight increase in deviation pattern between ~0.38 nm and ~0.41 nm respectively. Whereas, the mutants p.V68M and p.S188F, exhibited similar deviation pattern between ~0.42 nm and 0.45 nm, which is slightly higher than the native and mutant p.N126D. However, the complete convergence was observed for all the molecules at the end of 30 ns (Fig. 3a). In Root Mean Square Fluctuation (RMSF) analysis higher residual fluctuation of ~0.9 nm was observed in the mutant p.V68M. Whereas the native and other mutant proteins showed a similar fluctuation with maximum of ~0.7 nm (Fig. 4a). To verify the above results, the number of intramolecular hydrogen bonds formed within the protein was calculated using g_hbond (Fig. 5a). Among these mutants, p.N126D participated in the higher number of hydrogen bonds formation, followed by p.S188F and p.V68M respectively. The mutant p.I48T showed a constant number of hydrogen bonds formation. Solvent Accessible Surface Area (SASA) of the protein suggested that there is a loss of balance in the hydrophilic nature of the mutant p.S188F with a lesser SASA value (Fig. 6a), the mutant p.V68M showed higher SASA values, and the mutant p.I48T showed similar SASA values as that of the native. These results suggest that the mutants’ p.S188F and p.V68M exhibited higher structural differences from that of native protein when compared to the other mutants.

### Unique mutations

Similar analysis was performed for the unique mutations by comparing the mutants with the native protein. The RMSD plots predicted the mutant p.R246L with a higher RMSD of ~0.45 nm~0.42 nm at 30 ns. Whereas, the other mutants and native protein showed similar minimal deviation over the period of 30 ns (Fig. 3b). As for the common mutations, the convergence was observed at the end of 30 ns simulation for the unique mutations. From the RMSF plots, residual fluctuation in all the mutants was similar to that of native with a fluctuation of ~0.75 nm, except mutant p.R246L exhibited highest residual fluctuation of 0.85 nm (Fig. 4b). Further, number of intramolecular hydrogen bonds formed within the protein was analysed. The mutant p.R246L was involved in the least participation towards hydrogen bonds formation, followed by p.S135T. The p.Q307P mutant protein participated in higher number of intramolecular hydrogen bonds formation when compared to the native. On the other hand, the mutant p.S179N showed a similar number of intramolecular hydrogen bonds formation as of the native protein (Fig. 5b). Finally, SASA analysis was carried out for all the mutant proteins where the mutant p.R246L showed decreased SASA values, which further indicate that the mutant p.R246L could have lost contact with surrounding solvent molecules (Fig. 6b). Based on the above observation we conclude that the mutant p.R246L exhibited larger structural differences among the four unique mutations.

## Discussion

This present study focuses on the mutations that are causing the G6PDD among Arab populations (Fig. 2 and Supplementary Table S1). A comparison between the mutations in Arabs and Asians showed that most of the mutations responsible for the G6PDD shared among the two ethnic groups, which could be due to the long history of admixture among the two ethnic groups. These mutations were also shared with other ethnic groups, for example, the most frequent mutation among Arabs, p.S188F, was also frequently reported in Greece, southern Italy, Spain, Bulgaria, Romania, Turkey, and Israel^14^,^15^,^16^,^17^. We identified the most frequent mutations (p.S188F, p.V68M, p.I48T and p.N126D) and unique mutations (p.N135T, p.S179N, p.R246L, and p.Q307P) circulated among Arabs. Analyzing the effects of each mutation on the protein’s structure and function is very crucial in large scale analysis which is laborious and time-consuming by experimental methods. In recent years many studies have focused on the importance of *in silico* prediction methods in analyzing the effects of mutations on protein function^18^,^19^,^20^,^21^. Generally, these methods make their predictions based on sequence and structured based information using physiochemical properties and are benchmarked by the curators with the known datasets and performed well^22^,^23^. Subsequently, in the current study, we have used 5 *in silico* tools to estimate the pathogenic effect of the mutations and further compared with the WHO severity classification scale as a reference. The *in silico* prediction tools reported p.S188F, p.R246L, and p.Q307P mutations to be severe, whereas the p.I48T, p.V68M, and p.N126D mutations as mild, which were in consistent with the WHO classification for Class II and Class III severity scale for the G6PD mutations, respectively. The p.N135T and p.S179N mutations were predicted to affect the function of the G6PD enzyme (Supplementary Table S1) but were not assigned to any WHO classification (Table 1). The p.N126D and p.V68M mutations belong to the same haplotype and therefore always inherited together, causing a deleterious effect on the function of the G6PD enzyme^24^.

**Figure 7:**
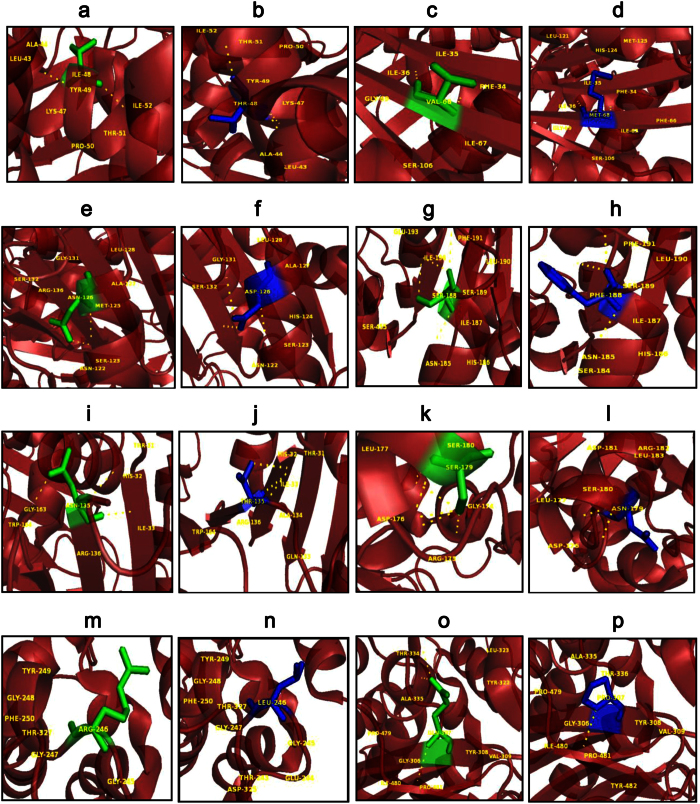
PyMOL visualization to compare the native and mutant amino acids. (**a**) native I48, (**b**) mutant p.I48T, (**c**) native V68, (**d**) mutant p.V68M, (**e**) native N126, (**f**) mutant p.N126D, (**g**) native S188, (**h**) mutant S188F, (**i**) native N135, (**j**) mutant p.N135T, (**k**) native S179, (**i**) mutant p.S179N, (**m**) native R246, (**n**) mutant p.R246L, (**o**) native Q307, (**p**) mutant p.Q307P. This visualization helps to predict the possible changes occurred upon mutation. Major differences observed are discussed below. In p.I48T, there is gain of polar contacts with Tyr49 and Leu43. Gain of contact with a hydrophobic amino acid such as Leucine further changes the orientation of the amino acid. P.V68M, where there the mutant (M) is larger than the native (V). In p.S188F, there is loss of polar contact indicating loss of stability. Loss of contacts are also observed in p.N125T mutant. Arginine (R) at 246^th^ position is in surface, further substitution with hydrophobic amino (L), destabilizes the protein structure. In mutant p.Q307P, a larger amino acid is substituted with much smaller Proline (P) and forming contacts with Tyr 482 (polar amino acid) changes the orientation of the mutant amino acid.

An extensive study that combines and elucidates the results from a systematic search with structural analysis helps in understanding the impact of mutations on much broader perspectives like protein stability and flexibility. The protein structure stability plays a predominant role in maintaining the functionality of a protein^25^. A mutation can affect the protein stability (both destabilizing and over stabilizing) leading to deterioration of the protein function through physico-chemical properties changes of the mutant amino acids (charge, size, hydrophobicity, hydrogen bonds)^26^. In recent years, MDSA has proved to be a powerful tool in elucidating the changes in a macromolecule at an atomistic level, thereby rendering better prediction results^27^,^28^,^29^,^30^. In this context, we segregated the common and unique mutations circulated among Arab patients with G6PDD to analyze the potential mutational impact on the function of G6PD enzyme using MDSA and local surrounding residual changes within 4 Å (Fig. 7a–p). Consequently, various analyses for the trajectories were performed to support our findings. The mutants p.S188F and p.V68M showed the highest deviation followed by the mutant p.N126D in the RMSD plots (Fig. 3a); a higher RMSD value predicts a decrease in the stability of protein structure^31^. To analyse the possible reason behind the change in the stability, other parameters such as the number of intramolecular hydrogen bonds formation, and SASA were elucidated. While calculating the number of intramolecular hydrogen bonds formed within the protein, reduction in a number of hydrogen bonds formations was observed in the mutant p.S188F (Fig. 5a). The decrease in hydrogen bonds in the mutant p.S188F can be due to the substitution of the amino acid with different physiochemical properties: serine being a polar amino acid actively participates in hydrogen bond formation, subsequently this nature is lost due to a substitution with a non-polar amino acid phenylalanine (Fig. 7g and h). Polar amino acids are commonly present in the exposed regions of the protein; any mutations in this region are very likely to affect the protein’s function^32^. Since S188 is present in the exposed region (Supplementary Fig. S3), we assessed both serine and phenylalanine contribution towards solvent interaction using SASA analysis (Fig. 6a), interestingly we observed a reduction in the SASA value in the presence of phenylalanine compared to serine, explaining a loss in the contact with the surrounding solvent which may further interfere with the functioning of p.S188F mutant protein. Similarly, in case of mutant p.V68M, the substituted amino acid methionine is a larger amino acid (M.W.: 131.21 Da) than the native amino acid valine (M.W.: 99.14 Da) and found to be located on the beta-sheets of the protein (Fig. 7c and d). Beta-sheets are known to be with rich hydrogen bonds between the protein strands, therefore mutations that occur in the beta-sheets are likely to interfere with hydrogen bond formation, which was observed in the mutant p.V68M (Fig. 5a). We also found deviations with the mutant p.I48T, whereas isoleucine, a hydrophobic amino acid, tends to orient towards the interior of the protein molecule, whereas, threonine a hydrophilic amino acid tends to lie in the outer region of the protein. Hydrophobic residues most often reside in the buried region of the protein, leading to a larger gain in stability than the burial of hydrophilic residues^33^. This decrease in stability was observed with higher RMSD values (Fig. 3a). Notably, most of the disease-related mutations were found to have an effect on the stability rather than the functioning of the protein^34^. Substitution of isoleucine (hydrophobic) in the buried region with threonine (hydrophilic) might induce unfavourable interaction with the neighboring hydrophobic amino acid (leucine 43) leading to a change in folding patterns (Fig. 7a and b). In the mutant p.N126D, aspartic acid introduces a negative charge, which allows the formation of hydrogen bonds with other nearby positively charged amino acids and consequently, increase in number of hydrogen bonds formation (Fig. 7e and f) which showed correlation with MDSA (Fig. 5a).

The unique mutation p.R246L was the most deleterious and exhibited the highest RMSD values (Fig. 3b) with few hydrogen bonds formation (Fig. 5b). Arginine is a hydrophilic amino acid that tends to be on the surface of the protein. A change in hydrophobic to hydrophilic nature of an amino acid in the buried region might lead to stability change and function of the protein^35^. Arginine interacts with the solvent and increases the stability; further substitution with a hydrophobic amino acid (leucine) might leads to destabilization which were consistent with findings from RMSD and hydrogen bond analysis (Figs 3b and 5b). In the mutant p.Q307P, glutamine, a polar amino acid present on the surface, is replaced by the substitution of a non-polar (proline) amino acid, which leads to the structural changes^36^. Polar amino acids present on the surface tend to interact with the surrounding solvent molecules. Substitution with proline induce interactions with polar amino acid tyrosine (Tyr482), subsequently changes the interaction pattern of the protein (Fig. 7o and p). The other two mutants, p.N135S, and p.S179N show similar dynamic activity as that of the native protein, which were not reported by the WHO classification. Hence from the observed MDSA results and the physiochemical changes, we conclude that the p.S188F (common) and p.R246L (unique) mutations may have an effect on the stability of the protein.

## Conclusion

In summary, we have comprehensively collected, systematically analysed, and elucidated the structural changes that occurred upon mutations in G6PD using high end computational approach. We mapped all the mutations circulated in Arab patients with G6PDD through systematic search of four different databases. We found 33 mutations (23 missense mutations, one silent mutation, two deletions, and seven intronic mutations); of these, the most frequent mutations are p.I48T, p.V68M, p.N126D, and p.S188F, which are shared with Asians and Europeans, and the mutations p.N135T, p.S179N, p.R246L and p.Q307P were unique to Arabs. It is suspected that there are more mutations that are unique to Arabs to be discovered, due to the prevalence of consanguineous and endogamous marriage among Arabs and the considerable number of undiagnosed patients due to the lack of comprehensive health care system, which gives Arabs a distinctive genetic profile compared with other ethnicities in terms of susceptibility to G6PDD. A systematic review has gained importance in clinical healthcare settings to understand the plausible mutations present in a population. This study demonstrates the usefulness of combination of the structural and computational analysis in understanding the genotype-phenotype correlation of the disease and paves the way for the application of user friendly tools in variant assessment in clinical molecular genetic diagnostics.

## Methods

### Study Selection

A systematic search was performed using four databases (PubMed, Embase, ScienceDirect, and Web of Science) to capture the circulated mutations among Arab patients with G6PDD in the 22 Arab countries from inception to May, 2016. The studies were then selected based on the following criteria: (1) published as a primary research paper in a peer-reviewed journal, (2) only Arab patients residing in Arab countries, and (3) Arab patients that were diagnosed with G6PDD. Combinations of search terms were restricted to “G6PD deficiency” OR “Glucose-6-phosphate deficiency” OR “G6PDD”, together with the name of each Arab country individually OR the terms: “Gulf” OR “GCC” OR “Arab”.

### *In silico* predictions

The pathogenicity of the 23 missense mutations among the Arab population was assessed using five *in silico* prediction tools, namely, SNAP2^37^ (https://www.rostlab.org/services/snap/), SIFT^38^ (http://sift.jcvi.org), PolyPhen-2^39^ (http://genetics.bwh.harvard.edu/pph2/), SNPs&Go^40^ (http://snps-and-go.biocomp.unibo.it/snps-and-go/), and Mutationassessor^41^ (http://mutationassessor.org/).

### Statistical analysis

A statistical analysis was performed to measure the consistency of the *in silico* prediction tools using parameters such as PPV (positive predictive value), NPV (negative predictive value), sensitivity/TPR (true positive rate), specificity/TNR (true negative rate), FPR (false positive rate), FNR (false negative rate), ACC (accuracy), and MCC (Matthews correlation coefficient). Mutations with deleterious scores predicted by the *in silico* tools were designated as ‘positive’ and those with non-deleterious scores were designated as ‘negative’. True Positive (TP), True Negative (TN), False Positive (FP), and False Negative (FN) were calculated for each mutation by comparing the tool results and the prevalence, which was also used to calculate the above mentioned parameters for the 5 *in silico* tools. The reliability of the prediction can be determined by Matthews correlation coefficient (MCC), where a score of 1 indicates the best reliability, −1 indicates the worst reliability and a score near to 0 indicates that the prediction is a result of chance^42^. Based on the results obtained from the *in silico* tools, the mutations that could have an effect on the protein were selected. Furthermore, the mutants with the most deleterious effect and higher prevalence in Arab countries were categorized as common and unique mutations.

### Structure and conservation analysis

The crystal structure of the protein was retrieved from the Protein Data Bank (PDB) with ID: 2BHL^43^ and mutation analysis was performed using SwissPDB Viewer. A cross-species multiple sequence alignment (MSA) was performed for the G6PD sequence from mice, rat, hamster, *Bosindicus* (Bosin), and human. The ConSurf conservation tool was used to precisely assign conservation scores for each amino acid across species^44^.

### MDSA

The protein structures of the native and mutant were used as the initiation of Molecular Dynamics Simulation. The simulation for the protein structure was performed using Gromacs 4.5.6^45^ package with GROMOS96 43a1 force field^46^. Initially, the protein structure was solvated in a cubic box with 10 Å radii. Further, using “genion” tool, the system was neutralised by adding Chlorine ions as there was an overall positive charge. Subsequently, the system was energy minimised until a lowest energy of 1000 kJ was obtained. This energy minimised system was equilibrated by subjecting to NVT and NPT for 50000 steps each. Finally, a production MD step was performed for 30 ns (nanoseconds). Van der Waals interactions were modelled using 6–12 Lennard-Jones potentials, with a 1.4 nm cut-off. The long-range electrostatic interactions were calculated using the PME method, with a cut-off for the real space term of 0.9 nm. Covalent bonds were constrained using the LINCS algorithm^47^. The time step employed was 2 fs, and the coordinates were saved every 2 ps for analysis, which was performed using standard GROMACS tools. The resultant trajectories were then subjected to analysis with the help of utilities available in the Gromacs package. g_rms, g_hbond, g_rmsf, and g_sas were used to calculate the root mean square deviation (RMSD), number of hydrogen bonds formed, root mean square fluctuation (RMSF), and solvent accessible surface area (SASA), respectively. The results of the analysis were graphically represented using the GRACE software. PyMOL^48^, a molecular visualization tool was also used for representing structural changes of the protein.

## Additional Information

**How to cite this article**: Doss, C. G. P. *et al.* Genetic Epidemiology of Glucose-6-Phosphate Dehydrogenase Deficiency in the Arab World. *Sci. Rep.* **6**, 37284; doi: 10.1038/srep37284 (2016).

**Publisher’s note:** Springer Nature remains neutral with regard to jurisdictional claims in published maps and institutional affiliations.

## Supplementary Material

Supplementary Information

## Figures and Tables

**Table 1:** *In silico* prediction result and their corresponding WHO classification of common and unique mutations in G6PD.

Type	Mutants	Predicted Deleterious by *in silico* tools	WHO classification
Common mutations	p.I48T	SNPs&GO, PolyPhen-2, and Mutationassessor	Class III (Mild)
	p.V68M	SNPs&GO, PolyPhen-2, Mutationassessor, and SNAP2	Class III (Mild)
	p.N126D	None of the tools predicted effect	Class III (Mild)
	p.S188F	SNPs&GO, PolyPhen-2, SNAP2, and SIFT	Class II (Severe)
Unique mutations	p.N135T	SNAP2, and Mutationassessor	Not Reported
	p.S179N	SNPs&GO, PolyPhen-2, Mutationassessor, SNAP2, and SIFT	Not Reported
	p.R246l	SNPs&GO, PolyPhen-2, Mutationassessor, SNAP2, and SIFT	Class II (Severe)
	p.Q307P	SNPs&GO, PolyPhen-2, Mutationassessor, SNAP2, and SIFT	Class II (Severe)
