# Corrigendum: Genetic Epidemiology of Glucose-6-Phosphate Dehydrogenase Deficiency in the Arab World

**DOI:** 10.1038/srep39370

**Published:** 2017-01-03

**Authors:** C. George Priya Doss, Dima R. Alasmar, Reem I. Bux, P. Sneha, Fadheela Dad Bakhsh, Iman Al-Azwani, Rajaa El Bekay, Hatem Zayed

Scientific Reports 6: Article number: 3728410.1038/srep37284; published online: 11 17 2016; updated: 01 03 2017

The original version of this Article contained an error in the title of the paper, where “Genetic Epidemiology of Glucose-6-Phosphate Dehydrogenase Deficiency in the Arab World” was incorrectly given as “Genetic Epidemiology of Glucose-6-Dehydrogenase Deficiency in the Arab World”. This has now been corrected in the PDF and HTML versions of the Article.

