# Supplementary material for: Genetic Epidemiology of Glucose-6-Phosphate Dehydrogenase Deficiency in the Arab World
Source: Sci Rep. 2016 Nov 17;6:37284. doi: 10.1038/srep37284 (PMC5112515; doi:10.1038/srep37284)
Supplement: Supplementary Information [file srep37284-s1.pdf]

Genetic Epidemiology of Glucose-6-Dehydrogenase Deficiency in the Arab World

George Priya Doss C [1], Dima R. Alasmar [2], Reem I Bux [2], Sneha P [1], Fadheela Bakhsh [2], Iman Al-Azwani [2], Rajaa El Bekay [32], Hatem Zayed* [2].

1. Department of Integrative Biology, School of Biosciences and Technology, VIT- University, India

2. Department of Health Sciences, Biomedical Program, Qatar University, Doha, Qatar.

3. Laboratory of Biomedical Research, Virgen de la Victoria Clinical University Hospital, Málaga, 29010, Spain.

*Correspondence to:

Hatem Zayed, PhD, College of Health Sciences, Department of Biomedical Sciences, Qatar University, Doha, Qatar, hatem.zayed@qu.edu.qa

Phone: 00974-4403-4809

Fax: 00974-4403-1351

Supplementary Information

Supplementary Tables

Supplementary Table S1: Missense mutations in the *G6PD* gene along with *in silico* prediction and WHO classification.

Supplementary Table S2: Statistical evaluation of *in silico* prediction methods for the missense mutations in G6PD

Supplementary Table S3: Most common missense mutations in the Arab world for *G6PD* gene

Supplementary Figures

Supplementary Fig. S1: Flow diagram of selected articles.

Supplementary Fig. S2: Results from Clustal Omega for Multiple Sequence Alignment

Supplementary Fig. S3: ConSurf results predicting the range of conservation of the amino acids in the G6PD protein sequence

Supplementary Tables

Supplementary Table S1

NCBI rs IDs	AA Change	Countries	WHO Severity /Class	SNPs&GO (Functional Effect & Reliability Index)	PolyPhen-2 (Functional Impact & score)	Mutation assessor (Functional Impact & score)	SNAP2 (Functional Effect & Reliability Index)	SIFT (Tolerance Index & score)	References
rs76645461	p.I48T	Algeria, Tunisia, Iraq, Jordan, Kuwait, Saudi Arabia & UAE	Mild/III	D(1)	PD(0.985)	L(1.365)	E(5)	IT(0.00)	1-18
rs1050828	p.V68M	Tunisia, Iraq, Comoros, Oman, Jordan, Kuwait, Palestine, Saudi Arabia & UAE	Mild/III	D(0)	PD(0.999)	M(3.365)	E(60)	IT(0.04)	1-6, 9, 12-17, 19, 21-26, 28, 34, 35, 39 & 40
rs1050829	p.N126D	Algeria, Tunisia, Comoros, Iraq, Jordan, Kuwait, Oman, Palestine, Saudi Arabia & UAE	Mild/III	N(3)	B(0.0)	N(0.535)	N(-26)	T(0.43)	1-6, 9, 12-17, 19, 21-26, 28, 29 & 39
NA	p.N135T	Egypt, Palestine & Jordan	NR	D(2)	PD(0.998)	M (2.83)	E(22)	IT(0.00)	9, 17 & 22
rs137852313	p.E156K	Algeria	Mild/III	N(1)	B(0.005)	N(0.49)	N(-89)	T(0.29)	32
NA	p.S179N	Palestine	NR	D(7)	*PD(0.9)	H (4.485)	E(17)	IT(0.00)	21,22
rs5030872	p.D181V	Algeria & Tunisia	Severe/II	N(6)	B (0.008)	N(-0.29)	N(-90)	IT(0.01)	1, 3, 29, 31 & 32
rs5030868	p.S188F	Algeria, Tunisia, Bahrain, Comoros, Iraq, Jordan, Egypt, Kuwait, Oman, Palestine, Saudi Arabia	Severe/II	D(5)	B(0.022)	M(3.42)	E(84)	IT(0.00)	1-6, 9, 12-17, 20-27, 32-35, 38 & 40-43

		& UAE							
NA	p.M212V	Saudi Arabia	Mild/III	D(3)	PD(0.986)	M (2.685)	N(-79)	IT(0.00)	7,8, 10, 11, & 37
rs137852328	p.R227L	Algeria	Mild/III	D(9)	PD(0.988)	H(4.2)	E(68)	IT(0.00)	30
NA	p.R246L	Tunisia	Severe/II	D(9)	PD(0.997)	H(4.65)	E(85)	IT(0.00)	1,3 & 34
rs137852318	p.D282H	Algeria	Mild/III	D(2)	PD(0.995)	M(2.57)	N(-9)	IT(0.00)	12
rs137852327	p.V291M	Saudi Arabia	Severe/II	D(7)	PD(1.00)	H (4.52)	E(32)	IT(0.00)	17
NA	p.Q307P	Tunisia	Severe/II	D(9)	*PD(0.951)	H(4.64)	E(88)	IT(0.00)	1, 3 & 12
rs76723693	p.L323P	Tunisia & Saudi Arabia	Mild/III	D(8)	B(0.249)	M(3.105)	E(93)	T(0.09)	1,3 & 36
rs5030869	p.A335T	Algeria, Iraq, Jordan, Kuwait, Oman & Saudi Arabia	Severe/II	D(1)	PD(0.961)	L(1.83)	E(89)	T(0.16)	2, 4-6, 9, 13-16, 24 & 32
rs137852321	p.R387H	Palestine	Severe/I	D(3)	PD(1.00)	M (2.46)	E(34)	IT(0.00)	22 & 41
NA	p.K407T	Tunisia	Severe/II	D(9)	PD(0.998)	H(4.64)	E(82)	IT(0.00)	1, 3 & 12
NA	p.S448G	Saudi Arabia	NR	D(4)	*PD(0.454)	M (2.77)	E(12)	T(0.44)	11
NA	p.R454C	Saudi Arabia & UAE	Severe/II	D(8)	PD(1.00)	H(3.655)	E(72)	IT(0.00)	7,8, 10, 11, 21, 25, 26, & 37
rs72554665	p.R459P	Algeria	Severe/II	D(8)	PD(0.979)	M(2.67)	N(-48)	IT(0.00)	17
rs72554665	p.R459L	Tunisia	Severe/II	D(5)	*PD(0.729)	L(1.7)	E(55)	IT(0.00)	1, 3 & 12
rs72554664	p.R463H	Tunisia	Severe/II	D(5)	PD(0.994)	M(3.25)	E(74)	IT(0.00)	1, 3 & 12

*D- Deleterious, N-Neutral, B- Benign, M-Mild, L-Low, H-High, E- Effect, *PD-Possibly Damaging, PD- Probably Damaging, IT- Intolerant, T-Tolerant, NA-Not applicable & NR-Not reported. The information for the above table was retrieved through a systematic search using 4 different databases: PubMed (http://www.ncbi.nlm.nih.gov/pubmed), Embase (https://www.elsevier.com/solutions/embase-biomedical-research), ScienceDirect (http://www.sciencedirect.com/) & Web of Science (http://wokinfo.com/webtools/browsersearch/)

Supplementary Table S2

Statistical parameters	SNPs&GO	PolyPhen-2	Mutation-assessor	SNAP2	SIFT
TP	14	12	11	11	13
TN	6	6	6	6	5
FP	3	5	6	6	4
FN	0	0	0	0	1
Total	23	23	23	23	23
PPV	0.82	0.70	0.64	0.64	0.76
NPV	1	1	1	1	0.83
Sensitivity/TPR (%)	1	1	1	1	0.93
Specificity/TNR (%)	0.67	0.54	0.50	0.50	0.55
FPR	0.33	0.45	0.50	0.50	0.45
FNR	0	0	0	0	0.07
ACC (%)	0.86	0.78	0.74	0.74	0.78
MCC	0.74	0.62	0.57	0.57	0.54

TP-True positive, TN-True negative, FP-False positive, FN-False negative, TPR-True positive rate, TNR-True negative rate, FPR-False positive rate, FNR-False negative rate, ACC- Accuracy, & MCC- Matthews correlation coefficient.

Supplementary Table S3

rs IDs	Nucleotide change	AA change	Exon	Origin	Frequency
rs5030868	c.563C>T	p.S188F	Exon 6	Mediterranean	56.80%
rs1050828	c.376G>A	p.N126D	Exon 5	Africa	10.03%
rs76645461	c.143T>C	p.I48T	Exon 3	Algeria	5.51%
rs1050828	c.202G>A	p.V68M	Exon 4	Africa	10 %

Supplementary Figures

Supplementary Fig. S1

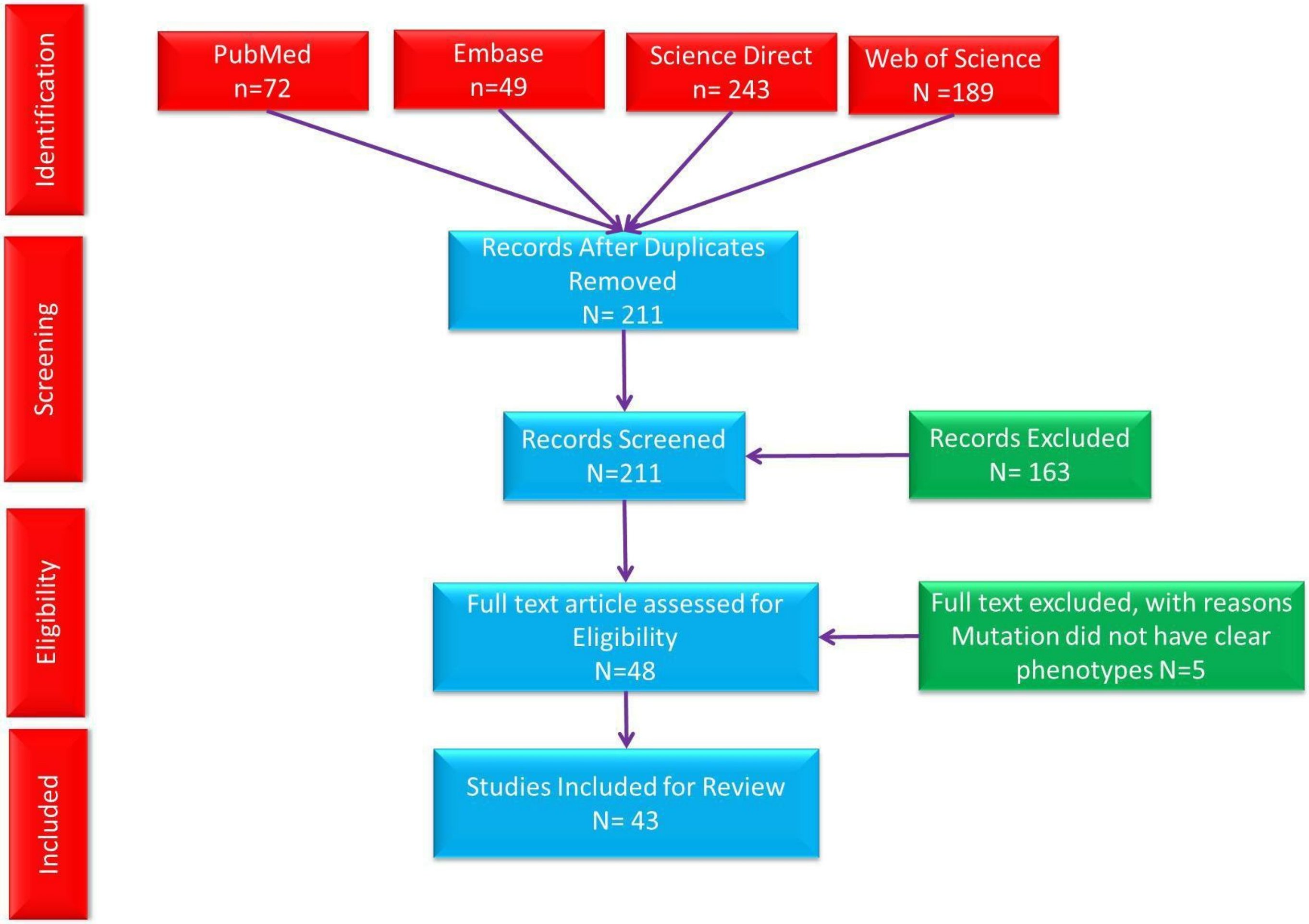

Supplementary Fig. S2

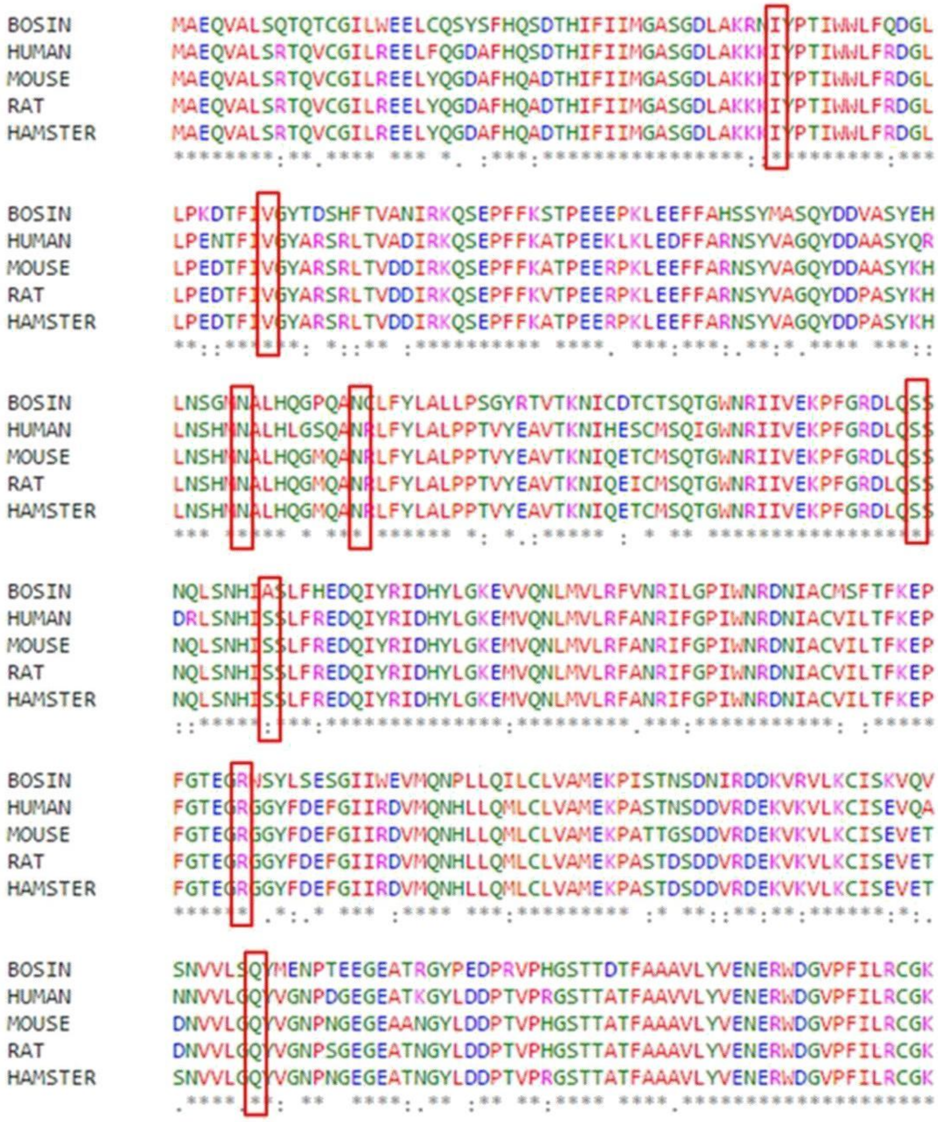

Supplementary Fig. S3

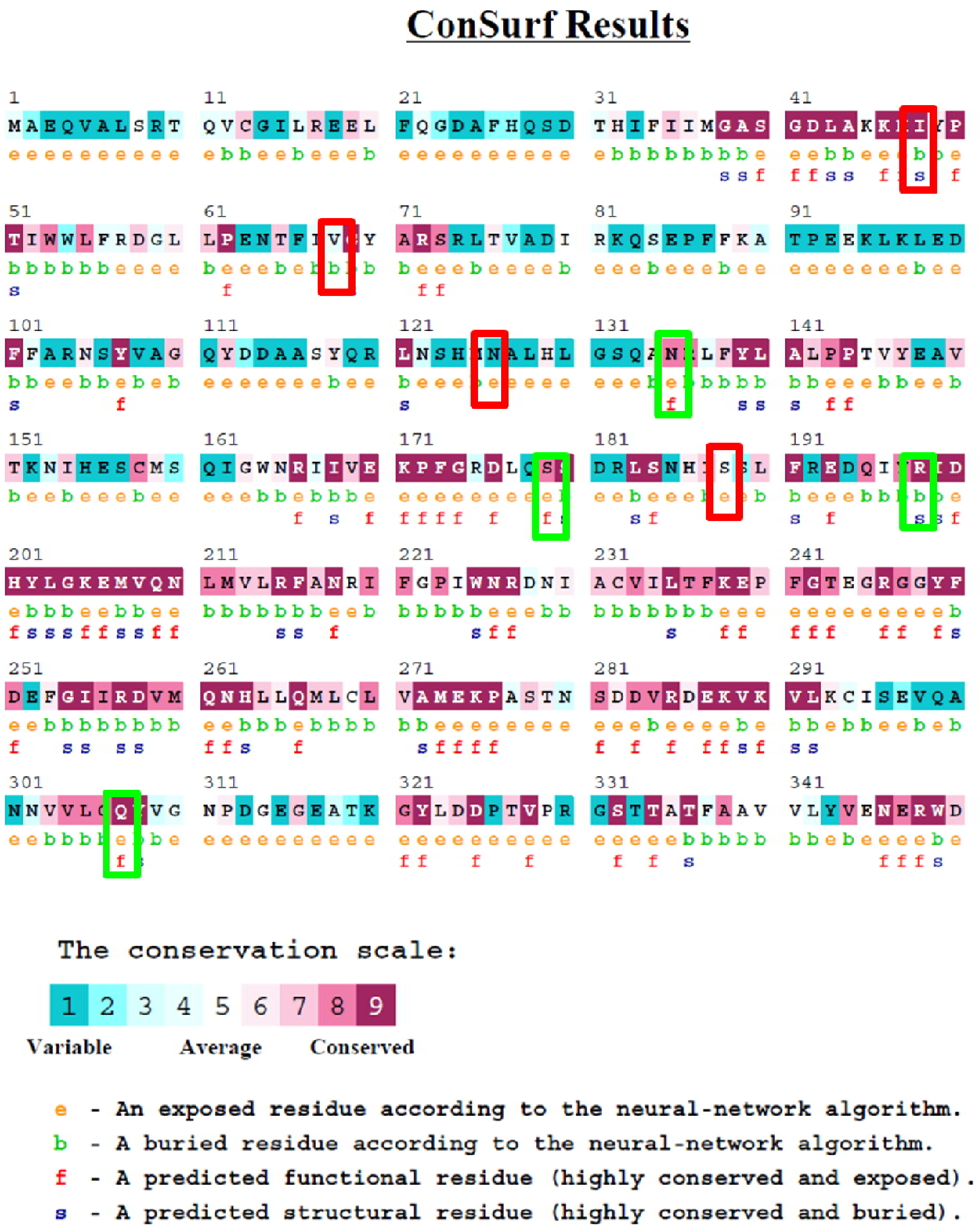

Supplementary References:

1. Bendaoud, B. *et al*. Three new mutations account for the prevalence of glucose 6 phosphate deshydrogenase (G6PD) deficiency in Tunisia. *Pathologie Biologie*. **61**(2), 64-69 (2013).

2. Al-Musawi, B. M. *et al*. Molecular characterization of glucose-6-phosphate dehydrogenase deficient variants in Baghdad city-Iraq. *BMC Hematology*. **12**, 4 (2012).

3. Benmansour, I. *et al*. Two new class III G6PD variants [G6PD Tunis (c. 920A> C: p. 307Gln> Pro) and G6PD Nefza (c. 968T> C: p. 323 Leu> Pro)] and overview of the spectrum of mutations in Tunisia. *Blood Cells, Molecules, and Diseases*. **50,** 110-114 (2013).

4. Al-Allawi, N. *et al*. Prevalence and molecular characterization of Glucose-6-Phosphate dehydrogenase deficient variants among the Kurdish population of Northern Iraq. *BMC Hematology*. **10,** 6 (2010).

5. Al-Alimi, A. *et al*. Mediterranean glucose-6-phosphate dehydrogenase (G6PD (C563T)) mutation among Jordanian females with acute hemolytic crisis. *J. Coll. Physicians Surg. Pak*. **20**, 794-797 (2010).

6. AlFadhli, S. *et al*. Molecular characterization of glucose-6-phosphate dehydrogenase gene defect in the Kuwaiti population. *Archives of pathology & laboratory medicine*. **129,** 1144 (2005).

7. Al-Jaouni, S., Al-Muhayawi, S. & Qari, M. Glucose-6-Phosphate Dehydrogenase Deficiency Correlation between Genotype and Phenotype. *Medical Science*. **14,** (2007).

8. Al-Jaouni, S. K. *et al*. Molecular characterization of glucose-6-phosphate dehydrogenase deficiency in Jeddah, Kingdom of Saudi Arabia. *BMC research notes.* **4,** 436 (2011.).

9. Al-Sweedan, S. A. & Awwad, N. Molecular characterization of glucose-6-phosphate dehydrogenase deficiency among Jordanians. *Acta haematologica*. **128,** 195-202 (2012).

10. Niazi, G. *et al*. Aures: A rare mutant of G6PD in Saudi Arabia. Molecular and clinical presentations. *Saudi medical journal*. **17,** 311-314 (1996).

11. Mohamed, M. M. & El-Humiany, A. U. R. Molecular characterization of new variants of glucose-6-phosphate dehydrogenase deficiency gene isolated in Western Province of Saudi Arabia causing hemolytic anemia. *Pakistan Journal of Biological Sciences*. **9**, 1605-1616 (2006).

12. Daoud, B. B. *et al*. Molecular characterization of erythrocyte glucose-6-phosphate dehydrogenase deficiency in Tunisia. *Pathologie Biologie*. **56,** 260-267 (2008).

13. Karadsheh, N. S. *et al*. Molecular heterogeneity of glucose-6-phosphate dehydrogenase deficiency in Jordan. *Haematologica*. **90,** 1693-1694 (2005).

14. D'Souza, B., Samilchuk, E. & Al-Awadi, S. Molecular analysis of the G6PD-Mediterranean mutation in Kuwait. *Medical Principles and Practice*. **7**, 209-214 (1998).

15. Samilchuk, E. *et al*. Glucose-6-phosphate dehydrogenase (G6PD) mutations and UDP-glucuronosyltransferase promoter polymorphism among G6PD deficient Kuwaitis. *Blood Cells, Molecules, and Diseases.* **31,** 201-205 (2003).

16. Kashmoola, M. A. *et al*. Molecular Characterization of G6PD Deficient Variants in Nineveh Province, Northwestern Iraq. *Indian Journal of Hematology and Blood Transfusion*. **31**, 133-136 (2015).

17. Amro, S. A. B. *et al*. Molecular Characterization of Glucose-6-Phosphate Dehydrogenase Deficiency in Abu Dhabi District, United Arab Emirates. Tropical. *Journal of Pharmaceutical Research*. **13**, 731-737 (2014).

18. Tadmouri, G. O., Sastry, K. S. & Chouchane, L. Arab gene geography: From population diversities to personalized medical genomics. *Glob. Cardiol. Sci. Pract*. **2014** (4)**,** 394-408 (2014).

19. Alharbi, K. K. & Khan, I. A. Prevalence of glucose-6-phosphate dehydrogenase deficiency and the role of the A- variant in a Saudi population. *J. Int. Med. Res.* **42**, 1161–1167 (2014).

20. Kurdi-Haidar, B., Mason, P.J., Berrebi, A., Ankra-Badu, G., al-Ali, A., Oppenheim, A. & Luzzatto, L. Origin and spread of the glucose-6-phosphate dehydrogenase variant (G6PD-Mediterranean) in the Middle East. *Am J Hum Genet.* **47**(6), 1013-9 (1990).

21. Sirdah, M. *et al.* Molecular heterogeneity of glucose-6-phosphate dehydrogenase deficiency in Gaza Strip Palestinians. *Blood Cells, Molecules, and Diseases*. **49**, 152-158 (2012a).

22. Sirdah, M. et al. Hemolysis and Mediterranean G6PD mutation (c. 563 C> T) and c. 1311 C> T polymorphism among Palestinians at Gaza Strip. *Blood Cells, Molecules, and Diseases* **48**, 203-208 (2012b).

23. Badens, C. *et al*. Molecular basis of haemoglobinopathies and G6PD deficiency in the Comorian population. The Hematology Journal **1,** 264-268 (2000).

24. Daar, S. *et al*. Molecular characterization of G6PD deficiency in Oman. *Human heredity,* **46,** 172-176 (1996).

25. Bayoumi, R. *et al*. Molecular characterization of erythrocyte glucose-6-phosphate dehydrogenase deficiency in Al-Ain District, United Arab Emirates. *Human heredity* **46,** 136(1996).

26. Dawodu, A. *et al*. Epidemiology of clinical hyperbilirubinaemia in Al Ain, United Arab Emirates. *Annals of tropical paediatrics*. **18**, 93 (1998).

27. Arnaout, H. H., El-gharbawy, N. M. Shaheen, I. & Afifi, R. Incidence and Association of 563 C / T Mediterranean and the Silent 1311C / T G6PD Mutations in G6PD-deficient Egyptian Children. *LabMedicine*. **42,** 355–360 (2011).

28. Benabadji, M. *et al*. Heterogeneity of glucose-6-phosphate dehydrogenase deficiency in Algeria. *Human genetics*. **40**, 177-184 (1978).

29. Usanga, E. A. & Ameen, R. Glucose-6-phosphate dehydrogenase deficiency in Kuwait, Syria, Egypt, Iran, Jordan and Lebanon. *Human heredity*. **50,** 158-161 (2000).

30. Blibech, R. *et al*. Incidence of glucose-6-phosphate dehydrogenase (G6PD) deficiency in Tunisian populations. *Nouvelle revue française d'hématologie*. **31**, 189-191(1988).

31. Nafa, K. *et al*. G6PD Aures: a new mutation (48 lle→ Thr) causing mild G6PD deficiency is associated with favism. *Human molecular genetics*. **2,** 81-82 (1993).

32. Nafa, K. *et al*. At least five polymorphic mutants account for the prevalence of glucose-6-phosphate dehydrogenase deficiency in Algeria. *Human genetics*. **94**(5), 513-517 (1994).

33. Hellani, A., Al-Akoum, S. & Abu-Amero, K.K. G6PD Mediterranean S188F codon mutation is common among Saudi sickle cell patients and increases the risk of stroke. *Genet Test Mol Biomarkers*. **13,** 449-52 (2009).

34. Abdulrazzaq, Y. M. *et al*. Diversity in expression of glucose 6 phosphate dehydrogenase deficiency in females. *Clinical genetics*. **55,** 13-19 (1999).

35. Samilchuk, E., D'Souza, B. & Al-Awadi, S. Population study of common glucose-6-phosphate dehydrogenase mutations in Kuwait. *Human heredity*. **49,** 41-44 (1999).

36. Minucci, A. *et al*. Glucose 6 phosphate dehydrogenase laboratory assay: How, when, and why? *IUBMB life*. **61**, 27-34 (2009).

37. Gari, M. *et al*. Frequency of Mediterranean mutation among a group of Saudi G6PD patients in Western region Jeddah. *International journal of laboratory hematology*. **32**, 17(2010).

38. Al-Ali, A. K. Common G6PD variant from Saudi population and its prevalence. *Ann Saudi*. Med **16** 654-656 (1996).

39. Hilmi, F.A., Al-Allawi, N.A., Rassam, M., Al-Shamma, G., & Al-Hashimi, A. Red cell glucose-6-phosphate dehydrogenase phenotypes in Iraq. *East Mediterr Health J*. **8**, 42-8 (2002).

40. Warsy, A. S. & El-Hazmi, M. F. G6PD deficiency, distribution and variants in Saudi Arabia: An overview. *Ann. Saudi Med*. **21,** 174–177 (2001).

41. Settin, A. *et al*. Screening for G6PD Mediterranean mutation among Egyptian neonates with high or prolonged jaundice. *Haema*. **9**83-90 (2006).

42. Al-Ali, A.K. *et al*. Molecular characterization of glucose-6-phosphate dehydrogenase deficiency in the Eastern Province of Saudi Arabia. *Clinical chemistry and laboratory medicine*. **40,** 814-816 (2002).

43. Al-Momen, N. Al Arrayed, S. S. & Al Allawi, A. Molecular homogeneity of GPD Deficiency. *Bahrain Medical Bulletin.* **26**, 1-7 (2004).
